# MEK Inhibition in Glioblastoma: Current Perspectives and Future Directions

**DOI:** 10.3390/ijms26146875

**Published:** 2025-07-17

**Authors:** Adam Shapira Levy, Jean-Paul Bryant, David Matichak, Shumpei Onishi, Yeshavanth Kumar Banasavadi-Siddegowda

**Affiliations:** 1 Department of Neurological Surgery, University of Miami Miller School of Medicine, Miami, FL 33176, USA; adam.levy@med.miami.edu; 2 Department of Neurosurgery, MedStar Georgetown University Hospital, Washington, DC 20007, USA; jean-paul.bryant@medstar.net; 3 Surgical Neurology Branch, National Institute of Neurological Disorders and Stroke, National Institutes of Health, Bethesda, MD 20892, USA; shumpei.onishi@nih.gov; 4 Department of Neurosurgery, University of Tennessee Health Science Center, Memphis, TN 38163, USA; dmatichak@uthsc.edu

**Keywords:** MEK, trametinib, glioblastoma, preclinical, clinical

## Abstract

The Mitogen-activated protein kinase kinase (MEK) protein family has dual-specificity protein kinases with a myriad of cellular functions that include but are not limited to cell survival, cell division, immunologic response, angiogenesis, and cellular senescence. MEK is crucial in the MAPK signaling pathway, regulating different organ systems, including the CNS. Increased activation and dysregulation of the MEK pathway is reportedly observed in 30% of all malignancies. The diversity of MEK renders it a prime target for inhibition in treating cancer. MEK inhibition has been studied in the context of melanoma, non-small cell lung cancer, breast cancer, and colorectal cancer, among others. The standard treatment for glioblastoma (resection, temozolomide, and radiation) remains relatively futile, which warrants alternative treatment options. Therefore, MEK inhibition has garnered more attention in recent years as investigators have explored its role in treating the most aggressive and most common primary brain tumor, glioblastoma. MEK inhibitors have shown efficacy in pre-clinical investigations as well as some promise in clinical trials which have demonstrated improved overall and progression-free survival. This underscores the potential of MEK inhibition in glioblastoma therapy and represents an area that likely warrants further research. However, there are few comprehensive and unifying reviews discussing the current state of MEK inhibition in glioblastoma therapy. We begin this review by detailing the normal function of MEK as it pertains to the CNS. We then compiled relevant pre-clinical and clinical studies to investigate recent research discussing the role of MEK inhibition in glioblastoma therapy.

## 1. Introduction

Mitogen-activated protein kinase kinase (MAP2K or MEK) represents a family of dual-specificity protein kinases with a range of cellular functions spanning cell survival and division, environmental stress, immunologic response, angiogenesis, and cellular senescence. MEK is a physiologically pervasive protein and plays a central downstream role in the MAPK signaling pathways (i.e., RAS-RAF-MEK-ERK) in numerous organ systems, including the central nervous system (CNS). Given the span of functions influenced by MEK, abnormal behavior of this kinase and its associated pathways has been implicated in carcinogenesis, with MEK alterations causing increased cell growth and proliferation, as observed in over 30% of all malignancies [1,2,3]. Given this prevalence, the development of MEK inhibitors has gained attention in recent years.

MEK inhibitors have shown promise in combating tumorigenesis as both monotherapy and combined therapy in several malignancies, including V600E melanoma, non-small cell lung cancer, breast cancer, and colorectal cancer [1]. Although the application of MEK inhibitors has been discussed for several conditions, the literature is devoid of a unifying review of MEK inhibitors and their therapeutic strategies for malignant conditions of the CNS, specifically that of glioblastoma. In this review, we describe the normal function of MEK in the CNS as well as its role in CNS malignancies. Further, we summarize existing clinical and pre-clinical investigations of MEK inhibitors in the treatment of glioblastoma.

## 2. Mitogen-Activated Protein Kinase (MAPK) Signaling Pathways

The MAPK signaling pathways, stimulated by a variety of growth factors, mitogens, and stress signals, regulate critical cellular functions including survival, proliferation, differentiation, apoptosis, and migration [3,4]. Among mammals, six unique MAPK pathways have been described: extracellular signal-regulated kinase (ERK)1/2, ERK3/4, ERK5, and ERK7/8; Jun N-terminal kinase (JNK) 1/2/3; and p38 isoforms α, β, γ, and δ [5,6,7,8]. Of these signaling pathways, four involve the enzyme activity of one of the seven known MEK (mitogen-activated protein kinase kinase) proteins, which we review below.

### 2.1. MEK 1,2: The ERK1/2 Pathway

**Figure 1:**
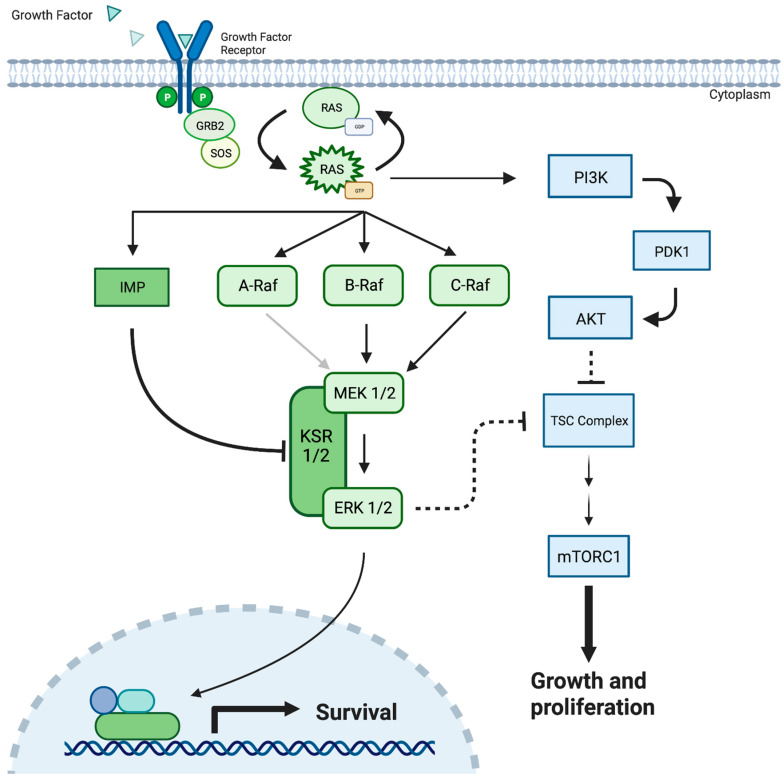
The MEK 1/2 pathway demonstrating cellular response to the growth factor protein, resulting in cellular survival, cellular growth, and proliferation. Mutated MEK/ERK can result in the uncontrolled cellular growth observed in cancerous solid tumors. MEK = Mitogen-Activated Protein Kinase; PDK-1 = Pyruvate Dehydrogenase Kinase-1; Akt = Protein kinase B; KSR = Kinase Suppressor of Ras; PI3K = Phosphatidylinositol 3-kinases; TSC = Tuberous Sclerosis Complex; GRB2 = Growth Factor Receptor-Bound Protein 2; SOS = Son of Sevenless Protein; IMP = Impedes Mitogenic Signal Propagation; RAS = Rat Sarcoma proteins.

The ERK1/2 cascade is the most studied MAPK pathway and begins with the binding of peptide growth factors to membrane-bound receptor tyrosine kinases such as epidermal growth factor receptor (EGFR), erbB2, platelet-derived growth factor receptor (PDGFR), and RET [9,10,11,12]. The subsequent dimerization and autophosphorylation of tyrosine residues facilitate the recruitment of the adaptor protein GRB-2 (growth factor receptor binding protein 2) and guanine nucleotide exchange factor SOS (Son of Sevenless) complex [13,14]. These factors mediate the conversion of Ras, a family of highly related proteins, H-Ras, N-Ras, K-Ras4A, and K-Ras4B from their inactive GDP-bound state to an active GTP-bound conformation [15]. Active Ras then facilitates the activation and recruitment of a family of three serine/threonine kinases, A/B/C Raf, to the plasma membrane [16] (Figure 1). Alternatively, many Raf-independent effectors of Ras have been identified, including Tiam1 GEF, phosphoinositide 3-kinases (PI3Ks), Ras-interaction/interference (RIN) proteins, and RalGEF, presenting a significant branch point in the cascade [15]. Interestingly, this branch point carries potential clinical implications, as it provides a potential escape mechanism where Ras mutations can bypass downstream MAPK/ERK pathway inhibitors, including MEK inhibition, to facilitate continued cell growth [13,15] (Figure 1).

As the ERK pathway continues, activated Raf enzymes then catalyze the ATP-dependent phosphorylation of MEK1/2 at serines 218 and 222 between the VII and VIII domains of the activation loop [3,17]. Additionally, the presence of a distinct hydrophobic pocket adjacent to the ATP binding site, capable of facilitating highly specific ATP-independent inhibition, has made MEK1/2 a favorable therapeutic target [18,19].

Raf isoforms have been found to vary in their abilities to activate MEK [3]. B-Raf is the strongest activator, likely secondary to a constitutive negative N-**terminus** facilitating improved binding and activation [3,20]. Additionally, Raf-MEK activation efficiency is thought to be further enhanced by the protein scaffolds KSR1/2 [21,22]. Raf A/B/C and MEK1/2 contain limited substrate specificities, with MEK1/2 serving as the only known Raf substrates and ERK1/2 serving as the only known MEK1/2 substrates [14].

Following MEK1/2 phosphorylation, ERK1/2 translocates to the nucleus, where its broad substrate specificity allows for the phosphorylation and activation of an array of transcription factors that influence cell growth and proliferation [13,23,24]. Aberrancy within this signaling cascade has been identified as one of the most frequently involved mechanisms of tumorigenesis, occurring in more than one-third of all malignancies [3,25].

### 2.2. MEK 4,7: The JNK Pathway

**Figure 2:**
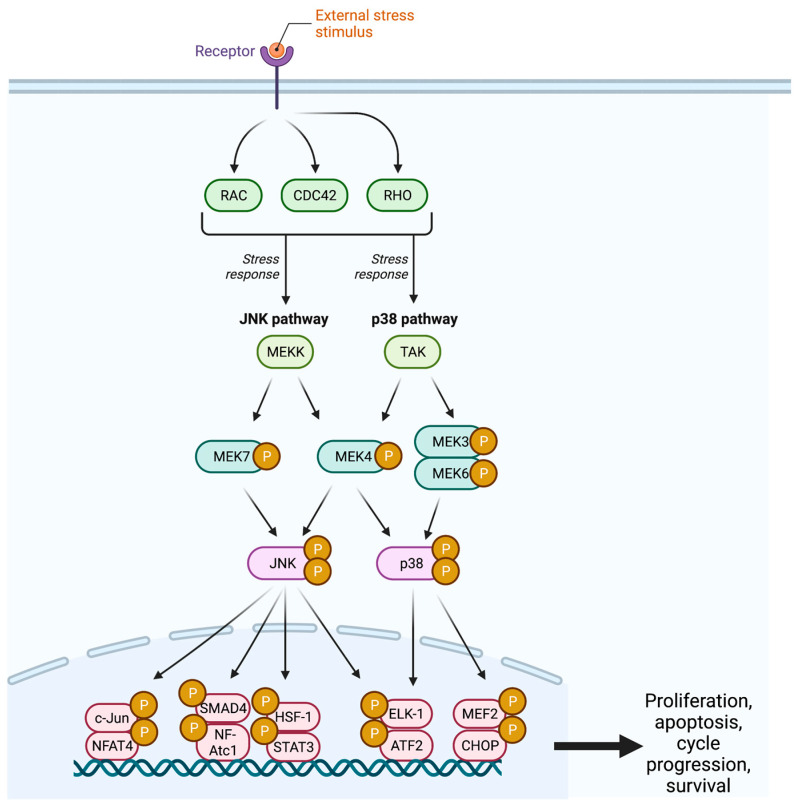
The JNK pathway demonstrates the cellular response to an external stress stimulus, resulting in cellular proliferation, cell cycle progression, and cellular survival. JNK = c-Jun N-Terminal Kinase; CDC42 = Cell Division Control Protein 42; MEK = Mitogen-Activated Protein Kinase; TAK = Transforming Growth Factor Β-Activated Kinase; SMAD4 = Mothers Against Decapentaplegic Homolog 4; NFAT = Nuclear Factor of Activated T Cells; HSF1 = Heat Shock Factor 1; STAT3 = Signal Transducer and Activator of Transcription 3; ELK1 = ETS-Like-1; ATF2 = Activating Transcription Factor 2; MEF2 = Myocyte Enhancer Factor 2; CHOP = C/EBP Homologous Protein; P = Phosphate.

The c-Jun NH2-terminal kinase (JNK) pathway serves as a stress signaling pathway and is activated by proinflammatory cytokines, UV light, DNA damage, growth factor deprivation, hyperosmolarity, and heat shock signals [26,27,28].The cascade begins with the GTPase activity of the Rho subfamilies Rho, Cdc42, and Rac, which target an array of MAPKK kinases (MAPKKK) including MEKK, MLK, TAK, ASK, and Tpl2 (Figure 2) [1,24]. These MAPKKKs facilitate the dual phosphorylation and subsequent activation of MEK4 and MEK7 at two sites within the activation loop (T-loop) [29].

MEK4, a ubiquitous cellular protein, is located on chromosome 17p11.2 and is expressed as a group of three alternately spliced isoforms [29,30,31,32]. MEK4 has been demonstrated to activate all mammalian JNK isoforms (JNK1, JNK2, and JNK3); however, as it contains containing N-terminus D-domain docking sites for both JNK and p38, it has been shown to also activate the p38 isoforms p38α and p38β [33,34,35,36]. Following their activation, JNK translocates to the nucleus where it participates in the phosphorylation of the transcription factors c-Jun, ELK-1, ATF2, NF-Atc1, HSF-1, and STAT3 [37].

The JNK pathway has been reported to play a suppressive role in tumor development and metastasis, with studies revealing a MEK4 loss-of-function mutation in approximately 5% of human tumors (such as breast, biliary, and pancreatic cancers) from a variety of tissues, and the identification of a microchromosomal region containing the *MEK4* gene revealed its ability to suppress cell metastasis [32,38,39,40]. However, findings also demonstrate JNK’s contributions to tumorigenesis within specific cellular contexts, as several studies have shown JNK facilitation of the oncogenic effects of Ras, epidermal growth factor, Met, BCR-Abl, and c-fos [40,41,42,43,44,45,46].

### 2.3. MEK 3,4,6: The p38 Pathway

Like the JNK pathway, the p38 pathway is a stress signaling pathway activated by DNA-damaging agents, environmental stimuli, and inflammatory cytokines (Figure 2) [7]. These pathways share a common start to their cascade with Rho, Cdc42, and Rac GTPase activation of a wide range of MAPKKKs, including TPL2, ASK1, MEKK, DLK1, TAO, and TAK [47,48,49]. Similar to the JNK pathway, MAPKKK diversity at this point in the pathway allows for improved processing and responsiveness to an array of external stimuli [50].

The p38 pathway has been demonstrated to regulate several cellular functions, including proliferation, apoptosis, cell cycle progression, and survival [51,52,53]. Like JNK, the *p38* gene has been previously described as a tumor suppressor; however, recent studies have revealed its potential roles in tumorigenesis as a promoter of stress resistance, survival, and cancer cell migration [47,54].

### 2.4. MEK 5: The ERK5 Pathway

The ERK5 cascade is the most recently identified of the MAPK pathways and follows a similar three-tiered kinase signaling model as ERK1/2, JNK, and p38 [55]. However, while the ERK1/2 and JNK/p38 pathways are stimulated by growth factors and stress signals, respectively, ERK5 is activated by both [56]. Its many stimulators include the mitogenic stimuli of EGF, PDGF, and VEGF and the stress stimuli of oxidative, osmotic, hypoxic, and proinflammatory cytokine signaling [57].

The cascade begins with the MEKK2 and MEKK3 phosphorylation of MEK5 at Ser311 and Thr315 [58,59]. Additionally, MEK5 activation has also been shown to be initiated by c-Src, TPL2/COT, AKT, and Ras/Raf [57,60]. This interaction demonstrates a continuation of the theme of cross-reactivity, as MEKK2 and MEKK3 have also been shown to stimulate the JNK and p38 pathways [61]. As a result of these intricate interactions, the MEK5/ERK5 pathway has been demonstrated to play a role in compensatory resistance to MEK1/2-ERK1/2 inhibitors [55]. Proposed mechanisms of resistance include increased RTK expression, the loss of ERK1/2 feedback inhibition of ERK5 and AKT, and enhanced TPL2/COT signaling [55,62,63,64,65].

In recent years, the MEK5/ERK5 pathway and its role in tumorigenesis have been extensively studied, and its influence has been demonstrated to be diverse [66,67,68,69]. Through numerous mediators, it has been proposed to influence key cancer processes such as angiogenesis, metastasis, immune evasion, and proliferation [70].

## 3. Pathophysiology of MEK Pathway and Role in Brain Tumorigenesis

As previously discussed, MEK plays a central role in the Raf-MEK-ERK pathway, serving as a major regulatory kinase of MAPK/ERK. Given the pathway’s importance in several fundamental cellular processes such as growth, differentiation, angiogenesis, migration, stress signaling, and apoptosis, much attention has been brought to the cascade and its role in disease, specifically cancer [71]. To date, dysregulation of the MEK pathway leading to increased cellular division and proliferation has been described to play a vital role in several broad categories of cancer, including melanoma, multiple myeloma, cholangiocarcinoma, pancreatic, lung, colorectal, breast, ovarian, testicular, and CNS tumors [3,72,73,74].

### 3.1. Upstream Dysregulation of MEK1/2

While MEK itself can become dysfunctional, the kinase is most often involved in tumorigenesis indirectly via upstream alterations in the Raf-MEK-ERK pathway, resulting in the hyperphosphorylation and activation of MEK1/2. This hyperactivation of MEK1/2 subsequently results in increased phosphorylation and activation of ERK1/2, a pair of serine/threonine kinases with >600 cytoplasmic and nuclear targets [75]. There are several well-documented upstream errors in this pathway. For example, EGFR overexpression due to both oncogenic mutations and defective receptor endocytosis has been observed to result in an autocrine signaling loop with consequent increases in cell proliferation, and it has been implicated in the pathogenesis of NSCLC, pancreatic, colorectal, glioblastoma, and triple-negative breast cancers [76,77]. Another common group of upstream errors are activating mutations in Ras and Raf family kinases, most notably BRAF and NRAS in melanoma and KRAS in pancreatic and colorectal cancer [78,79,80]. Activating mutations in other Ras (HRAS) and Raf (A-RAF and C-RAF) protooncogenes are similarly involved in tumorigenesis with subsequent upregulation of MEK/ERK, although observed less frequently due to additional regulatory constituents [81,82,83].

Alternative non-intrinsic MEK-activating pathways, that is, those not associated with EGFR/RAS/RAF mutations nor with mutations to MEK itself, have also been identified in select cancers such as uveal melanoma, where mutations in the Ras-like domain of the GPCR Gq subunit GNAQ drive the transcriptional activation of MEK [84].

### 3.2. Intrinsic Dysregulation of MEK 1/2

Recently, more attention has been brought to the dysfunction of MEK itself. Of the seven known mitogen-activated protein kinases (MEK1-7), MEK1 and MEK2 have been most extensively studied. Although less common than Ras- and Raf-driven cancers, oncogenic mutations in MEK have been described in increased tumorigenic pathway activation. Yuan et al. identified two classes of oncogenic mutations of MEK in a genomic database survey [85]. The group found a novel family of oncogenic in-frame deletions of the regulatory β3-αC loop of MEK1 to cause increased MEK homodimerization that leads to MEK1 activation, resulting in Raf-independent phosphorylation of ERK. In addition, the study corroborated the effect of inactivating mutations of the negative regulatory helix A region of MEK1, where mutation relieves the inactive conformation of the protein, resulting in increased kinase activity [85,86].

Several other MEK mutations have been observed in cancer formation and progression. In a recent pan-cancer analysis performed by Zhou et al., MEK1 was observed to have a relatively high mutation frequency with varying expression and functional impacts across >15 different types of cancer [87]. Among the 32 cancers analyzed, 60 oncogenic and likely oncogenic mutations in MEK1 were identified, some of which harbor known sensitivity to existing MEK1 inhibitors. In addition, 50 MEK1 mutations of unknown significance were identified by the authors, indicating an area for further investigation.

## 4. The Role of MEK3-7 in Cancer

Although less attention has been placed on MEK3-7, recent evidence suggests that the abnormal activation of these non-classical MEKs may play an equally important role in tumorigenesis, specifically with regard to the cellular stress response in cancer [88]. Unlike MEK1/2, MEK3 and MEK6 serve as regulatory kinases in the MEK-p38 axis, a tumor-suppressive pathway involved in the regulation of cell cycle transition, transcription, and apoptosis [89]. Reduced expression of MEK6 has been observed in several cancers, with its mechanism related to subsequent decreased activity of p38 and later p53, with reduced p53-mediated apoptosis and unchecked cell cycle inhibition [90]. The downregulation of MEK6 is also believed to play a role in radioresistance in select malignancies [91].

The MAPKKs MEK4/7 are involved in the stress-activated JNK/P38 pathway, with MEK4 acting on both JNK and P38 and MEK7 exclusively on JNK [22]. The inactivation of MEK4 is consistently observed in several malignancies, with downregulation frequencies as high as 75%, as in the case of serous ovarian cancer [92]. The exact mechanism of MEK4 in tumorigenesis is contentious given its effector JNK’s pro- and anti-oncogenic role; however most agree that it primarily functions as a tumor suppressor, with a significant reduction in survival observed in a variety of solid tumors with the loss of MKK4 [93,94,95]. The loss of MEK7 in carcinogenesis similarly disrupts the JNK cascade, leading to the dysfunction of a variety of cellular processes, including senescence, proliferation, and tumor metabolism, though its mechanism and specific role in these processes are not fully understood [96].

MEK5 is perhaps the least like its cousins, acting as the sole activator of the MAP kinase ERK5 in a mechanical stress-induced pathway [56]. It is currently believed that the MEK5-ERK5 pathway participates in a significant degree of cross-talk with the RAS-RAF-MEK1/2-ERK1/2 pathway, namely by acting as an “escape route” for tumors, such as melanoma, being treated with targeted MAPK inhibitor (MAPKi) therapy [97]. Research in this area is current and serves as a promising target for therapy-resistant malignancies. Interestingly, although much focus has been placed primarily on MEK5 in cancer, the MEK5-ERK5 pathway has been noted to play a substantive role in heart disease, angiogenesis, and neuronal survival [98,99].

## 5. MEK Involvement in Primary Brain Tumors and Glioblastoma

Primary sequence alterations have been identified in three major signaling cascades believed to be responsible for the development of glioblastoma. In a retrospective biospecimen genetic study performed by McLendon et al., the three pathways RTK-RAS-PI3K, p53, and RB were observed to be altered in 88%, 87%, and 78% of glioblastoma cases, respectively, with nearly 75% of the samples harboring alterations in all three cascades [100]. Although less common, the MAPK MEK-ERK1/2 pathway has been implicated in increased cell survival, migration, and invasion in glioblastoma, with direct upstream mutations in the MAPK pathway having been identified, namely in RAS and BRAF [101].

Among BRAF-associated glioblastomas, three distinct mutation classes have been described [102]. Class 1 accounts for V600E-mutated BRAF, an alteration that maintains the BRAF monomer in its active configuration, allowing for dimerization-independent activation of MEK1/2 [2]. Class 1 BRAF mutations have been described in 3% of adult glioblastoma and up to 20% of pediatric glioblastoma and are considered a potentially valuable target in recurrent glioma [103]. BRAF class 2 mutations, on the other hand, undergo RAS-independent dimerization with subsequent MEK1/2 activation [102]. While this mutational class is most frequently observed in low-grade gliomas, KIAA1549-BRAF fusions have been reported in rare cases of pediatric glioblastoma [104]. Class 3 BRAF mutations increase MEK-ERK activity via increased RAS-RAF binding and are commonly observed in conjunction with the loss of NF1 and/or RTK alterations [105]. While members of the mutation group have been observed in low-grade gliomagenesis, none have yet been described in glioblastoma [102].

Increased phosphorylation of the MEK1/2 effector ERK1/2 is associated with accelerated growth and invasiveness seen in high-grade gliomas [106]. Ramaswamy et al. demonstrated that inhibition of the MEK1/2-ERK1/2 pathway led to increased extracellular matrix (ECM)–cell adhesion within the glioblastoma microenvironment with resultant decreased proliferation and migration [107]. Although an in vitro study, these findings further suggest MEK to be an important entity in targeted therapy for glioblastoma.

Lastly, among the three primary subtypes of glioblastoma, upregulation of the MEK-ERK cascade has been most heavily implicated in the mesenchymal (MES) subtype. In a surrogate mouse model study, Marques et al. showed that the loss of NF-1 and the ensuing RAS-MEK-ERK upregulation is central in the high degree of stemness and plasticity observed in MES glioblastoma [108]. Furthermore, the group identified that MEK-ERK1/2 activation led to increase transcription factor FOSL1 activity, a critical component of glioblastoma mesenchymal transformation and cancer aggressiveness [108]. Although traditional pharmacological inhibition of targets such as FOSL1 would prove difficult, novel therapeutic avenues such as gene therapy could hold value. While the degree of MEK involvement has been less effectively characterized in glioblastoma tumorigenesis than other signaling proteins, the area is relatively understudied and may very well provide future value in the treatment of glioblastoma.

## 6. Pre-Clinical Applications of MEK Inhibitors for Glioblastoma Therapy

While the use of MEK inhibition in glioblastoma therapy is a relatively new topic in pharmacotherapy, there have been numerous studies in the pre-clinical setting examining the application of these agents on glioblastoma cell lines (Table 1). For example, our group was among the first to show that protein arginine methyltransferase 5 (PRMT5) inhibition in glioblastoma neurospheres responds to trametinib (MEK1/2 inhibitor) both in vitro and in vivo [109]. We demonstrated that PRMT5 depletion enhanced trametinib-induced cytotoxicity in glioblastoma as well as decreased trametinib-induced AKT and ERBB3 escape pathway activity (Table 1) [109]. Combination therapy with both PRMT5 depletion and trametinib prolonged overall survival in our intracranial glioblastoma xenograft mouse models. While we were the first to evaluate combination therapy with PRMT5 inhibition and trametinib, investigators have been examining the use of various combination therapies in conjunction with MEK inhibitors for glioblastoma therapy. For example, Essien et al. tested the combination of a histone deacetylase inhibitor (HDACi) and trametinib in conjunction with 4 Gy irradiation in glioblastoma stem-like cells (GSCs) [110]. In this in vitro study, the investigators found HDACi/MEK combination therapy to reduce both the activity of GSCs on the sphere formation assay and the level of expression of GSLC markers on flow cytometry. Moreover, the combination therapy more effectively reduced GSLC marker activity when compared to temozolomide and radiation therapy. Similarly, Schreck et al. examined the effect of combination therapy with the mTORC1/2 inhibitor sapanisertib and trametinib against glioblastoma-derived cell lines [111]. Sapanisertib was shown to act synergistically with trametinib by enhancing both the apoptotic and antiproliferative effects of trametinib in loss-of-function NF1 cell lines while also slowing the proliferation of MEKi monotherapy-insensitive cell lines. Combination therapy also showed an effect in cell lines insensitive to either monotherapy alone, suggesting sapanisertib/tramatenib therapy to be of potential benefit in glioblastoma patients lacking genomic predictors of RAS–effector dependence [111].

In addition to trametinib, several other MEK inhibitors have been studied in the preclinical setting. Shannon et al. studied the effects of PD0325901 (Mirdametinib), a second-generation MEK1/2 inhibitor, against glioblastoma cell lines (Table 1) [116]. Mirdametinib was shown to reduce growth rate on 2D and 3D cultures in previously unresponsive cell lines while also showing impedance of in vitro dispersal via a combination of changes in cell morphology and ECM properties. Likewise, Meskini et al. tested mirdametinib–PI3K inhibitor BKM120 combination therapy both in vitro and in vivo [119]. While monotherapy showed no significant improvement in the genetically engineered mouse (GEM) model, combined therapy resulted in a significant in vivo survival benefit and tumor growth inhibition. Tumor biomarker analysis of the group’s orthotopic model treated with combined therapy suggested that mirdametinib blocked feedback induction of the PI3K pathway’s effector activity on Ras-Raf-MEK while also suppressing the activity of the PI3K downstream target protein S6 (pS6), both reducing proliferation and enhancing apoptosis of tumor cells.

Separately, Paternot et al. studied combination therapy with mTOR inhibitor rapamycin and the small-molecule MEK1/MEK2 inhibitor PD184352 [120]. The group found that mTOR/MEKi combination therapy achieved near complete in vitro inhibition of DNA replication, retinoblastoma protein (pRB) ribosylation, and CDK4 ribosylation among glioblastoma cell lines. Stepanenko et al. investigated another potent small-molecule non-competitive MEK inhibitor, U0126, in combination with the mTOR inhibitor temsirolimus [118]. Temsirolimus/U0126 therapy was shown to cause chromosomal instability and increased sensitivity to glucose depletion in several glioblastoma cell lines, while other lines underwent significant proliferation upon exposure to the drug regimen. Further, a cell-lineage-dependent response was observed in colony formation efficiency, as well as a cell-line-dependent re-treatment sensitivity. The findings highlight an important mechanism of drug-mediated genotypic and phenotypic evolution of glioblastoma, with authors cautioning for a careful balance of genotoxicity when evaluating for the long-term success of chemotherapeutic agents [118].

McNeill et al. tested several MEK inhibitors of varying potency in combination with PI3K inhibitors against both in vitro and in vivo glioblastoma models [117]. MEK inhibitors trametinib, PD0125901, and selumetinib all showed dose-dependent efficacy against the patient-derived xenograft (PDX) in vitro model while concurrently showing the activation of proximal PI3K signaling via an alternate pathway. Conversely, in vitro PI3K inhibitor monotherapy resulted in the activation of PI3K-MEK independent pathways, namely RAS-RAF-MEK-ERK, with MEKi-PI3Ki combination therapy yielding a synergistic in vitro effect, corroborating the findings by Meskini et al. [117,119]. The study also echoed the importance of kinome profiling, identifying hyperactive MEK1 activity in one of the two kinome subtypes observed in patient-derived models, suggesting a highly viable target in a subset of patients [117,118]. Interestingly, the MEKi selumetinib alone showed the greatest efficacy on the orthotopic in vivo mouse model, with minimal inadvertent activation of the PI3K pathway. Poor combined therapy results were owed largely to the unfavorable CNS pharmacokinetics and dose–toxicity of the studied PI3K inhibitors, namely buparlisib. Ultimately, preclinical findings have warranted further investigation of MEK inhibitors, especially as combined therapy, in the management of glioblastoma. A summary of these inhibitors and their use in pre-clinical studies is summarized in Table 1.

## 7. Clinical Applications of MEK Inhibitors in HGG and Glioblastoma

Following its demonstrated effectiveness against BRAF V600E metastatic melanoma, investigations of combined MEK/BRAF inhibition have been expanded to include glioblastoma variants [121,122]. In a multicenter, open-label, single-arm, phase 2 basket trial by Subbiah et al., investigators examined the use of dabrafenib (BRAF inhibition) and trametinib (MEK 1/2 inhibition) in the setting of recurrent/progressive BRAF V600E-mutant gliomas (NCT02034110). Forty-five patients with high-grade gliomas (HGGs) were enrolled, with 31 meeting criteria for a diagnosis of glioblastoma [123]. Within the HGG subgroup, an overall response rate of 33% was observed, with three patients demonstrating a complete response and most responders experiencing prolonged durations of the response [114,124] (Table 2).

These results led to the development of additional clinical trials among BRAF V600E-mutant HGG patient populations, illustrating the clinical utility of MEK inhibition. One study was the NCT03973918 phase 2 trial examining the novel combination of binimetinib (MEK 1/2 inhibition) and encorafenib (BRAF inhibition). Here, Schreck et al. administered binimetinib and encorafenib continuously at 28-day intervals [125]. All five patients enrolled in the study either had HGGs or glioblastoma with a BRAF V600E mutation that was recurrent following one aspect of therapy (i.e., radiation, immunotherapy, or chemotherapy). The radiographic response rate was 60% with one complete response and two partial responses (99% and 98% volume reduction). Four patients out of the five patients studied demonstrated decreases in tumor volume of at least 70% on MRI. At the time of publication of their results, three patients had discontinued the treatment due to disease progression; one discontinued due to an adverse event; and one remained currently in the trial. Median progression-free survival was 9.4 months, and median survival was 14.6 months. Three patients died after participating in the trial (Table 2).

NCT03919071 is a phase 2 trial investigating dabrafenib and trametinib (D + T) use following radiation therapy. This study is currently active; however, it is no longer recruiting patients. The results have yet to be submitted for this trial (Table 2).

NCT04190628 is a phase 1 trial evaluating the safety of ABM-1310 (BRAF inhibitor) and its combination with cobimetinib (MEK 1/2 inhibitor). This study was completed, and while the final results have not been reported, interim analysis has shown that ABM-1310 either alone or in combination with cobimetinib was well tolerated without unexpected safety issues [127] (Table 2).

Lastly, NCT05798507 is an early phase 1 clinical trial testing the brain concentration level and safety of defactinib or avutometinib (VS-6766). Each of these agents inhibits a different pathway in glioblastoma proliferation; therefore, the investigators hypothesize that they may create a synergistic treatment effect. These kinase inhibitors target Raf (VS-6766) and FAK (defactinib), which aim to reduce these abnormally expressed proteins to their normal functional state.

Further supporting the clinical utility of MEK inhibition in glioblastoma are several case reports of successful D + T (dabrafenib + trametinib) therapy among BRAF V600E mutants. In two cases of recurrent glioblastoma with standard therapy failure, complete and stable MRI/clinical responses to D + T therapy were observed at 11 and 16 months, respectively [129,130]. Additionally, despite the ultimate development of treatment resistance at 7 months, substantial tumor regression and KPS functional improvement were observed at 3 weeks in a patient receiving first-line D + T therapy secondary to glioblastoma with significant leptomeningeal involvement [131]. In two additional cases of leptomeningeal spread, impressive clinical and radiographic improvements were observed in as little as 1 week from the initiation of D + T therapy. In the case of a 28-year-old female receiving first-line D + T following subtotal resection, remarkable improvements in speech, strength, and endurance were observed at one week. At four weeks, a near complete MRI resolution of leptomeningeal disease and a lack of parenchymal progression were seen before the ultimate recurrence of her disease and therapy discontinuation at 11 months [115]. In the case of a 24-year-old male receiving D + T + bevacizumab (added for additional edema control) following GTR and standard therapy failure, profound improvements were seen at one week as the patient was no longer wheelchair bound, walking independently, and experiencing resolution of aphasia [115]. At three months, leptomeningeal and parenchymal regression was observed on MRI before the patient became non-compliant with therapy and experienced a fatal recurrence [115]. Moreover, in the case of a 42-year-old male receiving D + T with silybin, a STAT3 inhibitor added due to the presence of a SOX2 mutation, a complete radiographic and metabolic response was achieved after 3 months and was sustained for 24 months after therapy initiation [132].

In a retrospective review aimed at profiling the natural history of 19 BRAF V600E mutant glioblastoma patients, 5 patients were found to have been treated with D + T. Within this cohort, a non-significant trend suggesting longer overall survival with BRAF/MEK inhibition was observed, demonstrating a median overall survival of 35.6 months compared to 17.0 months among those not receiving BRAF/MEK therapy [133].

### NF-1 Associated Glioblastoma

Among Neurofibromatosis type 1 (NF-1) patients, approximately 20% will develop primary CNS gliomas [134]. Despite low-grade gliomas (LGGs) being more common, these patients are also at risk for developing HGGs, for which the use of MEK inhibition therapy is sparse [135]. In a molecular analysis of 47 NF-1 glioma patients, 9 patients received MEK inhibitor therapy. Four out of the five LGG patients achieved stable disease, while three out of the four high-grade astrocytoma/glioblastoma patients experienced mass progression [136]. To date, only two case reports of MEK inhibitor therapy among NF-1-associated glioblastoma patients have been described. In a 19-year-old male receiving trametinib as a fourth-line treatment for NF-1-associated glioblastoma, a complete response was achieved at 10 months, which was noted to be sustained at a 13-month clinic visit [137]. Similarly, as a fourth-line treatment, trametinib therapy was provided to a 24-year-old male suffering from NF-1-associated glioblastoma on a compassionate care basis. At the 3-week mark, he demonstrated a reduction in mass enhancement and mass effect, with further MRI radiographic improvement at 2 months after treatment initiation [138].

## 8. Limitations/Drawbacks

Although early investigation has shown promise in the effect of MEK inhibitors in the treatment of glioblastoma with significant antiproliferative effects, the issue of compensatory resistance mechanisms and rebound aggressiveness among MEK-inhibited cell lines remains. Corroborating the findings of several studies cited in this review, Selvasaravanan et al. showed that MEKi therapy often leads to enhanced tumor mobility, an elevated epithelial-to-mesenchymal signature, and increased tumor invasiveness [139]. The phenomenon termed the “Go or grow” dichotomy is well documented in glioblastoma and is attributed to the notorious therapeutic difficulty associated with the disease [140].

Further, the development of MEK inhibitors suitable for the treatment of glioblastoma relies heavily on their ability to permeate the blood–brain barrier. Gampa et al., for example, described the MEK inhibitor E6201 to have an almost 18-fold increase in CNS receptor-bound substrate when compared to trametinib as monotherapy [141]. Conversely, Vaidhyanathan et al. identified that the CNS distribution of trametinib is heavily dependent on the efflux pump P-glycoprotein, and they noted that the brain–plasma distribution ratio increased by nearly ten-fold when combined with a BRAF inhibitor as dual therapy [142]. These studies highlight the need for the further investigation of the pharmacodynamics of both monotherapy and multitherapy with MEKi agents to maximize the delivery of the active agent to the site of active disease.

## 9. Conclusions

The unique tumor biology combined with the expansive genomic and kinomic profile of glioblastoma contributes to its difficulty in treatment. Advances in neuro-oncologic research are required in the development of novel therapeutic avenues, such as those of MEK inhibitors. Early investigations into MEK inhibition in glioblastoma therapy has shown some promise, with both in vitro and in vivo studies demonstrating some efficacy. Glioma stem cells have shown sensitivity to MEK inhibitors, which is critical for therapy as they are believed to contribute to treatment resistance. While clinical trials are in early phases, MEK inhibition with or without BRAF combination therapy has been shown to reduce tumor volume and influence survival in a limited number of patients. Additionally, narrowing down which MEK inhibitors are most effective in diminishing tumor burden will aid in creating a specific tumor treatment regimen. In the future, large-scale clinical trials are likely warranted to further investigate the efficacy of MEK inhibition in glioblastoma therapy. Continued investigations of these drugs are required to determine the reduction in disease burden and the progression of glioblastoma.

## Figures and Tables

**Table 1:** Summary of studies involving trametinib and other MEK inhibitors as monotherapy or combined therapy against glioblastoma. glioblastoma = Glioblastoma; PRMT5 = Protein Arginine Methyltransferase 5 (PRMT5); TMZ = Temozolomide; NF1 = Neurofibromatosis type 1; MEK = Mitogen-Activated Protein Kinase; TRP = Transient Receptor Potential; CDK4 = Cyclin-Dependent Kinase 4; HDAC = Histone Deacetylase; pRB = Retinoblastoma Protein.

Investigators (Year)	Treatment Method	Outcome
Khan et al. (2023) [112]	Trametinib	Four MEK inhibitors were screened, and trametinib demonstrated efficacy in induing apoptotic cell death in glioblastoma stem cells and thus were used for further experiments. Glioblastoma stem cells treated with trametinib in vitro promoted neuronal differentiation. Glioblastoma mouse models treated with oral trametinib had significantly improved survival (*p* < 0.01). A total of 25–30% had long-term survival.
Hornschemeyer et al. (2022) [113]	Dactolisib, ipatasertib, MK-2206, regorafenib, or trametinib	Human glioblastoma cells were treated with small-molecule inhibitors, including trametinib. Trametinib reduced proliferation, increased cell death, and interfered with signal transduction in two cell lines.
Banasavadi-Siddegowda et al. (2022) [109]	Trametinib and PRMT5 depletion	Combination therapy with both PRMT5 depletion and trametinib enhanced the cytotoxic effects of trametinib while reducing escape pathway activity. In vivo trametanib efficacy was likewise increased (*p* ≤ 0.001) when compared to the controls.
Essien et al. (2022) [110]	Trametinib, HDAC inhibition, and radiation therapy	Combined therapy resulted in in vitro inhibition of glioblastoma neurospheres. The effect was significant (*p* ≤0.001) when compared to TMZ ± radiation therapy across three glioblastoma cell lines.
Wen et al. (2021) [114]	Dabrafenib and trametinib	Thirty-one patients with glioblastoma were treated with combination therapy. A total of 12 out of the 31 patients exhibited partial tumor responses, and 3 out of the 31 patients exhibited complete tumor responses.
Schreck et al. (2020) [111]	Tramatenib and mTORC1/2 inhibition	Combined therapy induced apoptosis and growth inhibition in NF1-absent cell lines (*p* ≤ 0.001) when compared to the control and monotherapy with either agent alone. Combined therapy also showed an antiproliferative effect in MEKi-insensitive cell lines.
Johanns et al. (2018) [115]	Dabrafenib and trametinib	A patient with epithelioid glioblastoma demonstrated partial regression of the left frontal mass and profound symptomatic improvement (resolution of aphasia).
Shannon et al. (2017) [116]	Mirdametinib (PD0325901)	Mirdametinib reduced the in vitro growth rate of glioblastoma cell lines while reducing aggregation and cell dispersal.
McNeill et al. (2017) [117]	Trametinib/PD0125901/selumetinib and mTOR inhibition	MEK inhibition as a monotherapy showed efficacy against a glioblastoma xenograft model with concurrent dose-dependent activation of proximal PI3K escape pathway activity. Selumetinib monotherapy was the only treatment (*p* = 0.03) to improve in vivo survival in orthotopic TRP mouse models.
Stepanenko et al. (2016) [118]	U0126 and mTOR inhibition	Combined therapy showed a cell-line-dependent change in cell proliferation with varying degrees of ERK1/2 and AKT1 phosphorylation. A similar cell-line-dependent response was observed in re-treated cell lines.
El Meskini et al. (2015) [119]	Mirdametinib and PI3K inhibition	Combined therapy increased in vitro cell death while inhibiting > 70% more tumor growth when compared to the control in orthotopic modeling. In vivo overall survival was significantly greater (16 days, *p* < 0.001) when compared to both the control and monotherapy with either agent alone.
Paternot et al. (2009) [120]	PD184352 and mTOR inhibition	Combined MEK/mTOR therapy inhibited in vitro DNA replication and G_1_-S transition arrest via combined modulation of CDK4 and pRB phosphorylation.

**Table 2:** Summary of completed and existing registered clinical trials examining MEK inhibition in high-grade gliomas and glioblastoma. NCT = National Clinical Trial; NCI = National Cancer Institute; ABTC = Adult Brain Tumor Consortium; HGG = High-Grade Glioma; glioblastoma = Glioblastoma; MEK = Mitogen-Activated Protein Kinase Kinase.

Investigators (Year Started, Year Completed)	Clinical Trial ID	Treatment	Study Status	Pathology	Outcome
Subbiah et al. (2014, 2021) [123]	NCT02034110 (Phase II)	(BRAF inhibitor) and trametinib (MEK 1/2 inhibitor)	Completed	HGG	The HGG group had an overall response rate of 33%. Three patients demonstrated a complete response.
Schreck et al. (2019, 2023) [125]	NCT03973918 (Phase II)	Binimetinib (MEK 1/2 inhibition) and encorafenib (BRAF inhibition)	Terminated due to NCI terminating the ABTC consortium.	HGG or glioblastoma	Four out of the five patients with either HGGs or glioblastoma demonstrated at least a 70% reduction in tumor volume. Median progression-free survival was 9.4 months, and median survival was 14.6 months. Three patients died after participating in the trial.
Lulla et al. (2020, present) [126]	NCT03919071 (Phase II)	Dabrafenib (BRAF inhibitor) and trametinib (MEK 1/2 inhibitor)	Active, not recruiting	HGG	N/A
Piha-Paul et al. (2020, 2024) [127]	NCT04190628 (Phase I)	ABM-1310 (BRAF inhibitor) alone or in combination with cobimetinib (MEK 1/2 inhibitor)	Terminated (not related to safety/efficacy concerns)	glioblastoma	Interim analysis demonstrated that ABM-1310, either alone or in combination with cobimetinib, was well tolerated without unexpected safety issues.
Olson et al. (2023, present) [128]	NCT05798507 (Phase I)	Defactinib (FAK inhibitor) or avutometinib (Ras pathway inhibitor)	Recruiting	glioblastoma	N/A

## Data Availability

Not applicable.
